# Prevalence of chronic stress in general practitioners and practice assistants: Personal, practice and regional characteristics

**DOI:** 10.1371/journal.pone.0176658

**Published:** 2017-05-10

**Authors:** Anja Viehmann, Christine Kersting, Anika Thielmann, Birgitta Weltermann

**Affiliations:** Institute for General Medicine, University Hospital Essen, University of Duisburg-Essen, Essen, Germany; Azienda Ospedaliera Universitaria di Perugia, ITALY

## Abstract

**Background:**

The majority of studies investigating stress in primary care have focused either on general practitioners (GPs) or practice assistants (PAs), but did not measure stress on a practice level. We analyzed the prevalence of chronic stress for both professional groups and on a practice level and investigated personal, practice, and regional characteristics.

**Methods:**

Chronic stress was measured in GPs and PAs from 136 German practices using the standardized, self-administered TICS-SSCS questionnaire (12 items). Based on a sum-score, participants per professional group were categorized as having low or high strain due to chronic stress (≤ 25th and ≥ 75th percentile of the study population´s distribution, respectively). For a cluster-level analysis, the mean of all practice means was used to categorize low- and high-stress practices. The intra-class correlation coefficient (ICC) was calculated using ANOVA. Prevalence Ratios (PR) were used to compare low versus high strain due to stress, stratified for personal, practice and regional characteristics.

**Results:**

The response rate was 74.1% (n = 137/185). Data from 214 GPs (34.1% female), 500 PAs (99.4% female), and 50 PAs in training (98.0% female) were analyzed. Chronic stress was highest in female GPs (median 19, IQR (interquartile range) 11.5), followed by PAs (16, IQR 12.25) and male GPs (15, IQR 10). On a practice level, 26.3% of the practice personnel reported a high stress level. We observed an overall ICC of 0.25, with higher ICCs when stratifying by professional group (PAs: ICC 0.36, GPs in group practices: ICC 0.51). High chronic stress was observed as the number of working hours per week increased (GPs: PR 2.03, 95% CI 1.16–3.56; PAs: PR 2.02, 95% CI 1.22–3.35). There were no differences for practice type (solo/group) and the various regional characteristics.

**Conclusion:**

Personal and practice characteristics were associated with chronic stress in GPs, PAs, and on a practice level. The high ICCs indicate a need for stress-reduction strategies geared at both professions on a practice level.

## Introduction

While a number of studies have addressed stress among health care workers in the hospital setting [1–3], little research has addressed chronic stress among primary care professionals. Among these is the US Physician Worklife Study which conceptualized a stress model with personal, practice and patient characteristics as the key determinants for perceived stress in primary care [4]. Personal characteristics comprise, e.g., demographic characteristics (sex, age), working hours, and work experience, while examples for practice characteristics are practice size (solo/group practices; number of patients) and room facilities. The specific case mix of a practice (patient characteristics) is linked to the practice’s location [4] which is an indicator for regional characteristics, such as the sociodemographic structure and the economic-ecological context. Prior studies which compared urban and rural practice environments or analyzed associations of stress prevalence with community size yielded conflicting results [5–8].

So far, only few studies addressed all three determinants of stress. Research in this area is relevant not only regarding physician’s and other practice personnel’s health, but also from a patient perspective. Studies described a relationship between poor physician wellbeing and various patient outcomes such as low patient satisfaction, poor adherence to medical treatments, and a higher incidence of (serious) medical errors [9].

Of the few studies available, the majority focused on stress among general practitioners (GPs), while only few studies addressed chronic stress in practice assistants (PAs). Although primary care is typically provided by practice teams working together with the same patients, only two studies, one from Australia and one from Germany, addressed both professional groups simultaneously [7,8]. Both studies showed that GPs and PAs had a high level of job satisfaction. However, these studies did not carry out practice level analyses, even though these are important for a better understanding of the relationship between organizational characteristics and psychological outcomes.

This cross-sectional study attempts a more comprehensive approach by exploring stress (high versus low strain due to chronic stress) for both professions separately and for clusters of practice teams. We calculated the prevalence ratios for chronic stress in both professional groups and on a practice level. In addition, associations with personal, practice, and regional characteristics were investigated on an individual and practice cluster level.

## Methods

### Survey method and participants

All GPs and PAs of the 185 general medicine practices belonging to the practice network of the Institute for General Medicine, University Hospital Essen, Essen, Germany, were asked to participate in the study. The practices were located in urban and rural regions of North-Rhine-Westphalia (Western Germany) at an average distance of 30 km (range: 2–180 km) to the university hospital. In a prior study, we showed that our institute’s practice network is representative for German GP practices [10].

Data collection was carried out between April and September 2014 during on-site visits. Practices were invited by mail and contacted by phone for further recruitment communication. Those refusing to participate were asked to answer a short questionnaire on key practice characteristics and their reasons for non-participation. Within each practice, all GPs (practice owners and employed physicians) and practice assistants including medical secretaries and practice assistant trainees were eligible for participation and received the study documents. Study documents consisted of 1) an information sheet addressing data collection and storage including data protection issues, 2) an informed consent form to be completed by all participants prior to the survey, and 3) a self-administered questionnaire on chronic stress and working conditions. For data protection reasons, participants were asked to seal the completed questionnaire in an envelope. As an incentive, practice teams received a voucher of a department store chain (EUR 5 per person), irrespective of the participation of single team members.

Ethical approval was obtained from the Ethics Committee of the Medical Faculty of the University of Duisburg-Essen (reference number: 13-5536-BO, date of approval: 24/11/2014). All participants received written information and signed informed consent forms, which are stored at the institute. The ethics committee approved to this consent procedure.

### Measurement of chronic stress

The prevalence of chronic stress was assessed using the German short version of the Screening Scale of the Trier Inventory for the Assessment of Chronic Stress (TICS-SSCS), a psychometric questionnaire [11]. The TICS-SSCS is a standardized and validated instrument that measures strain due to chronic stress [11–13]. It captures five different domains of stress: chronic worrying, work-related overload, social overload, excessive demands, and lack of social recognition. Participants were asked to indicate frequencies for each of the 12 items on a five-point Likert scale (never = 0 points, rarely = 1 point, sometimes = 2 points, often = 3 points, very often = 4 points). The TICS-SSCS was chosen because it measures strain due to chronic stress retrospectively for three months rather than providing a snapshot. Moreover, it is suitable for both GPs and PAs and allows for a comparison of chronic stress in our study population with the German general population reported in the “German Health Interview and Examination Survey for Adults”(DEGS1) [14].

### Personal, practice and regional characteristics

For individual characteristics, participants were asked to complete a self-administered questionnaire addressing socio-demographic characteristics (e.g., age, gender, marital status), work-related characteristics, and items measuring chronic stress. Additionally, participants were surveyed on measures used to compensate for stress (e. g. sports, wellness, listening to music) and how frequently they use these (regularly, sometimes, rarely). Also, GPs and PAs were asked questions specific to their respective professional group.

For practice characteristics, practice owners were asked to provide information on the type of practice (solo/group), the number of practice team members differentiated by professional groups, the number of patients per three months, and the percentage of patients with statutory health insurance.

Regional data to characterize the practices’ locations were retrieved from public statistical data provided by the North-Rhine Westphalian government (http://www.statlas.nrw.de/Statlas/viewer.htm, 2015/12/16) [15]. This included data on the number of inhabitants, population density (inhabitants/km^2^), the percentage of non-German population, the percentage of elderly inhabitants (inhabitants ≥65 years of age), the unemployment rate (%), and the disposable income per capita and year (€). Data on road traffic noise (mean 24-hour noise levels) were extracted from noise maps with values for 2012 (http://www.umgebungslaerm-kartierung.nrw.de/, 2015/24/03) [16] which had been modeled and published according to EU Directive 2001/43/EC [17].

### Statistical analysis

All participants who answered the questions of the TICS-SSCS were included in the statistical analyses. Practice team members without patient contact and those working on a temporary basis (e.g. for an internship) were excluded from the analyses.

The TICS-SSCS values were added to a sum-score. The score ranges from 0 to 48, with 0 denoting “never stressed” and 48 “very often stressed”, and reflects subjective strain due to chronic stress.

Prevalence ratios (PR) and their 95% confidence intervals (CI) were calculated to compare participants and practices with low and high strain due to chronic stress. For the analysis at an individual level, the TICS-SSCS sum-score was categorized into three categories: low, medium, and high. For the assignment of participants to one of these categories, professional group-specific (GPs, PAs) and gender-specific cut-offs were calculated: using the ≤ 25^th^ and the ≥ 75^th^ percentile of the respective distribution of our study population, participants were categorized as having low and high strain due to chronic stress, while the 50% in-between these percentiles were categorized as having medium strain due to chronic stress. For male physicians, the TICS-SSCS cut-off points for the three categories were ≤ 9 low, 10–19 medium, ≥ 20 high; for female GPs the cut-off points were ≤ 13 low, 14–24 medium and ≥ 25 high. Because PAs were predominantly female, no gender-specific distribution was applied for this professional group: ≤ 10 low, 11–22 medium, ≥ 23 high. We used gender-specific and professional group-specific cut-offs, as it has been shown that age and gender as well as socio-economic status have an influence on the reporting behavior of the TICS-SSCS items [13,14]. To analyze personal determinants, prevalence ratios for low versus high strain due to chronic stress were calculated stratified by age, number of household members, marital status, professional experience (years in job; years in practice), working hours per week, employment status (practice owner/employed physician), and the number of measures used regularly to compensate for stress. These variables were dichotomized using the means for each professional group as cut-off.

For analyses at a cluster level (practice level), intra-cluster correlations (ICC) for practice teams, for GPs in group practices and for PAs [18] were calculated. For further practice-level analyses, practices were categorized into above-average and below-average practices: the mean of the practices’ TICS-SSCS mean values (≤ 16.6 / > 16.6) was used as a cut-off point. Practices with lower strain compared to those with higher strain due to chronic stress were analyzed stratified by type of practice (solo/group), caseload (number of patients per practice per three months) (median, ≤ 1,750 / > 1,750), percentage of patients with statutory health insurance (median, ≤ 85% / > 85%), number of consultation rooms (median, ≤ 3 / > 3), and mode of documentation (electronic / paper-based / combination).

This was performed likewise for the regional data, with the median values for the respective variable being used as a cut-off: number of inhabitants (≤ 220,000 / > 220,000), population density (≤ 2,095 / > 2,095 inhabitants/km^2^), percentage of non-German population (≤ 12.0% / > 12.0%), percentage of inhabitants aged > 65 years (≤ 21.4% / > 21.4%), unemployment rate (≤ 12.0% / > 12.0%), disposable income per capita and year (≤ 18,996 € / > 18,996 €) and the 24-hour mean road traffic noise (≤ 75 dB(A) / > 75 dB(A))

Statistical analyses were processed using IBM SPSS Statistics for Windows, Version 22 (Armonk, NY: IBM Corp.) [19]. The PRs with the 95% intervals were calculated using the R, package epiR and the procedure epi.2by2. Percentages and mean values are reported for valid cases.

## Results

The response rate was 74.1% (n = 137 practices) with n = 794 participants: 226 GPs and 568 non-physician practice team members. The non-responder analysis at practice level showed that there were no differences regarding key practice characteristics except that the proportion of solo practices was slightly higher in participants than in non-participants (39.3% versus 30.6%). The mean number of practice assistants (5.3 [SD 4.0] versus 4.7 [SD 2.7]), the mean number of GPs (2.3 [SD 1.5] versus 2.4 [SD 1.2]), and the quarterly caseload (median 1.750–2.000 per practice) were comparable between participating and non-participating practices.

We included data of n = 214 GPs and n = 550 PAs from 136 practices who were eligible for the analyses. 65.9% of the physicians were male (n = 141), while 98.5% of the PAs were female (n = 542) (Table 1). Male physicians were older (mean age: 53.9 years [SD 8.2]) than female physicians (47.6 [SD 8.2]). PAs were younger (mean age: 37.4 years [SD 12.7]) compared to the GPs. Regarding the marital status, 87.9% (n = 188) of the GPs and 50.6% (n = 277) of the PAs were married. We observed slightly more years in the job (work experience) in GPs (males 24.4 years [SD 8.8]; females 20.2 years [SD 9.3]) than in PAs (18.8 years [SD 12.5]). No male physician worked part-time, whereas this proportion amounted to 28.6% (n = 20) for female physicians and 33.5% (n = 179) for PAs. For details see Table 1.

**Table 1:** Characteristics of study participants (n = 764).

	Physicians		Practice assistants
	Male (n = 141; 65.9%)	Female (n = 73; 34.1%)	n = 550 (female: n = 542; 98.5%)
	n (%)	n (%)	n (%)
**Age** (mean, +/-SD)	53.9 (8.2)	47.6 (8.2)	37.4 (12.7)
**Physicians in GP training**	2 (1.4)	5 (6.8)	50 (9.1)
**Marital status**			
Single	7 (5.0)	9 (12.3)	218 (39.9)
Married	128 (92.1)	60 (82.2)	277 (50.6)
Divorced	4 (2.9)	4 (5.5)	45 (8.2)
Widowed	0 (0)	0 (0)	7 (1.3)
**Number of persons in household**			
Household members ≤3	94 (66.7)	51 (69.9)	444 (80.7)
Household members >3	47 (33.3)	22 (30.1)	106 (19.3)
**Working hours/week**			
≤39	21 (14.9)	31 (43.1)	403 (74.6)
40–59	82 (58.2)	34 (47.2)	127 (23.5)
≥60	38 (27.0)	7 (9.7)	10 (1.9)
**Employment status**			
Full-time	140 (100.0)	50 (71.4)	355 (66.5)
Part-time	0 (0.0)	20 (28.6)	179 (33.5)
Self-employed	134 (95.0)	51 (71.8)	-
Employed	7 (5.0)	20 (28.2)	-
**Work experience**			
Years in job (mean, +/-SD; IQR)	24.4 (8.8; 13.0)	20.2 (9.3; 15.0)	18.8 (12.5; 22.0)
Years in current practice (mean, +/-SD; IQR)	16.4 (9.3; 16.0)	10.1 (8.1; 13.0)	10.4 (9.3; 13.0)

Percentages and mean values are reported for valid cases.

The mean practice size was 2.3 GPs (SD 1.5) and 5.2 PAs (SD 4.0); 39.1% (n = 52) of the practices were solo practices (Table 2). 48.1% (n = 63) of practices provided medical care for up to 1750 patients per quarter (caseload). 54.5% (n = 72) of the practices worked with full electronic patient records, while the remaining 45.5% (n = 60) combined electronic and paper-based records. For details see Table 2.

**Table 2:** Practice characteristics (n = 136 practices).

	n	%
**Practice type**		
Solo practice	52	39.1
Group practice	81	60.9
**Patients per practice per quarter**		
≤ 1750	63	48.1
>1750	68	51.9
**Number of patients with statutory health insurance**		
≤ 85%	46	40.7
> 85%	67	59.3
**Number of treatment rooms**		
≤ 3	66	50.0
> 3	66	50.0
**Medical records**		
Paper-based	0	0
Electronic	72	54.5
Electronic and paper-based documentation	60	45.5

Percentages and mean values are reported for valid cases.

### High strain due to chronic stress per professional group and on cluster level

Of the 764 participants included in the analysis, 26.3% (n = 201) reported high strain due to chronic stress. Female physicians showed the highest median of the TIC-SSCS (median: 19, interquartile range (IQR) 11.5), followed by PAs (median: 16, IQR 12.25) and male physicians (median: 15, IQR 10). For comparison, a median of 11 was reported for the German general population [14]. When applying the DEGS1 cut-off for high stress (TICCS-SSCS ≥ 23) to our study population, 19.9% of the male physicians (n = 141) and 35.6% of the female physicians (n = 73) as well as 26.4% of the PAs (n = 550) showed high strain due to chronic stress.

On a cluster level, the mean TISC-SSCS value of all practice means was 16.6 (SD 4.9; range 4.0–31.7). Among the GPs (n = 214), 30.2% of those in single practices and 24.7% of those working in group practices reported high chronic stress. The proportions were also comparable among PAs: 27.9% of the PAs working in single practices and 25.9% of those in group practices reported high chronic stress. In single GP practices and group practices with at least one highly stressed physician, the proportions of PAs reporting high stress did not differ (30.0% versus 27.9%). We observed an overall ICC of 0.25. The ICC was higher when stratifying by professional group: among PAs we observed an ICC of 0.36, among GPs in group practices the ICC was 0.51.

### Personal characteristics

**Fig 1:**
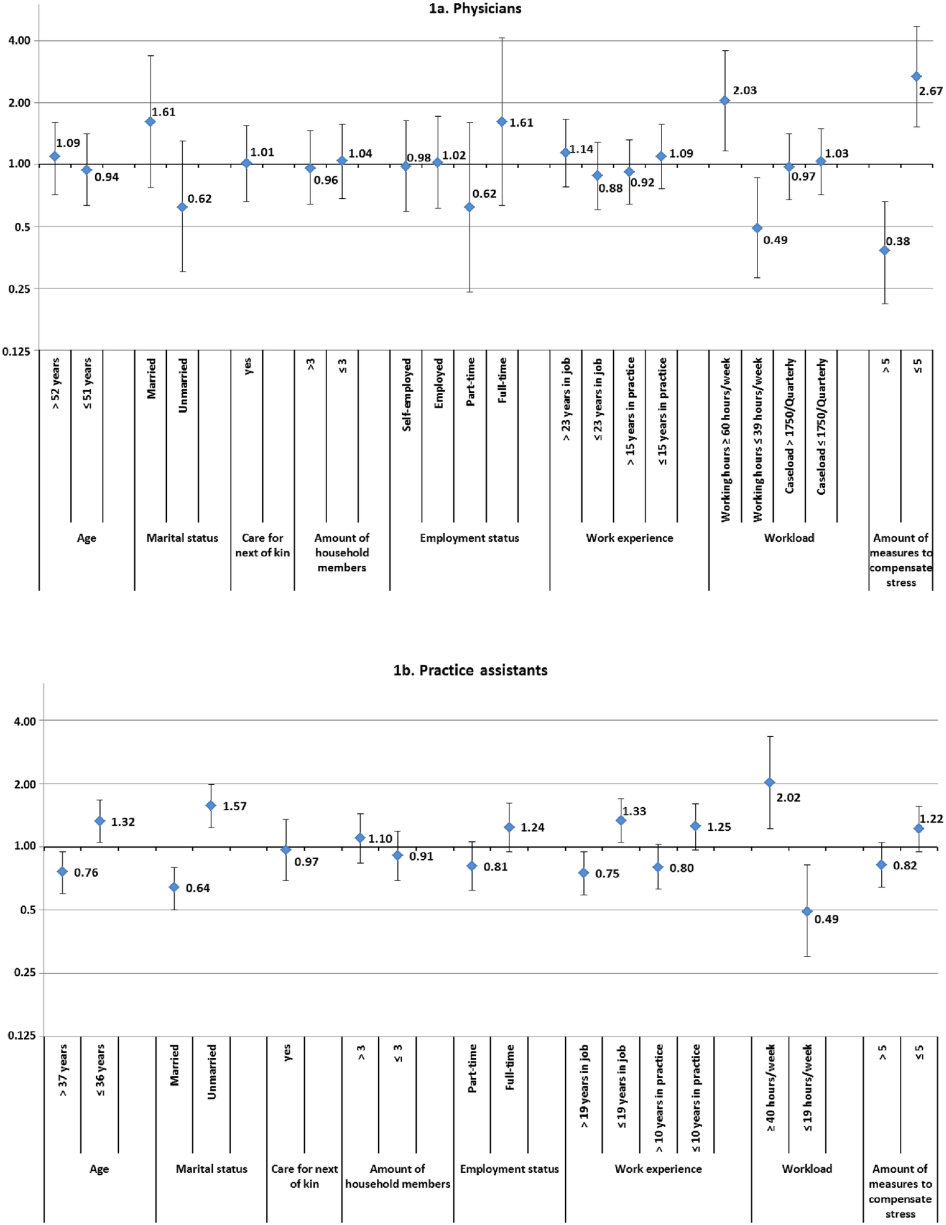
**1a. Physicians**. Prevalence ratios and 95% confidence intervals for low versus high strain due to chronic stress by personal characteristics. **1b. Practice assistants**. Prevalence ratios and 95% confidence intervals for low versus high strain due to chronic stress by personal characteristics.

In GPs, the prevalence ratios for age and for work experience (years in job; years in practice) did not show differences between subjects with low and high strain (Fig 1a). Physicians who were married had a higher PR than unmarried physicians (PR 1.61 [95% CI 0.77–3.38]). Regarding work-related characteristics, GPs working ≥ 60 hours per week had a 2.03-fold (95% CI 1.16–3.56) higher prevalence of high strain compared to those working ≤ 39 hours per week. Furthermore, GPs who individually applied more than five measures regularly to compensate for stress had a markedly lower PR (PR 0.38 [95% CI 0.21–0.66]).

In contrast to these results, we observed associations of strain due to chronic stress with age as well as work experience among PAs (Fig 1b). Younger PAs (≤ 36 years) showed higher strain due to chronic stress (PR 1.32 [95% CI 1.05–1.67]) than older PAs (> 37 years). Similar PRs were observed for years in practice and years in the job. Regarding work-related personal characteristics, PAs working ≥ 40 hours had a higher PR (2.02; 95% CI 1.22–3.35). Comparable to the results in GPs, we observed a PR of 0.82 (95% CI 0.64–1.05) in PAs who regularly applied more than five measures for stress compensation.

### Practice characteristics

**Fig 2:**
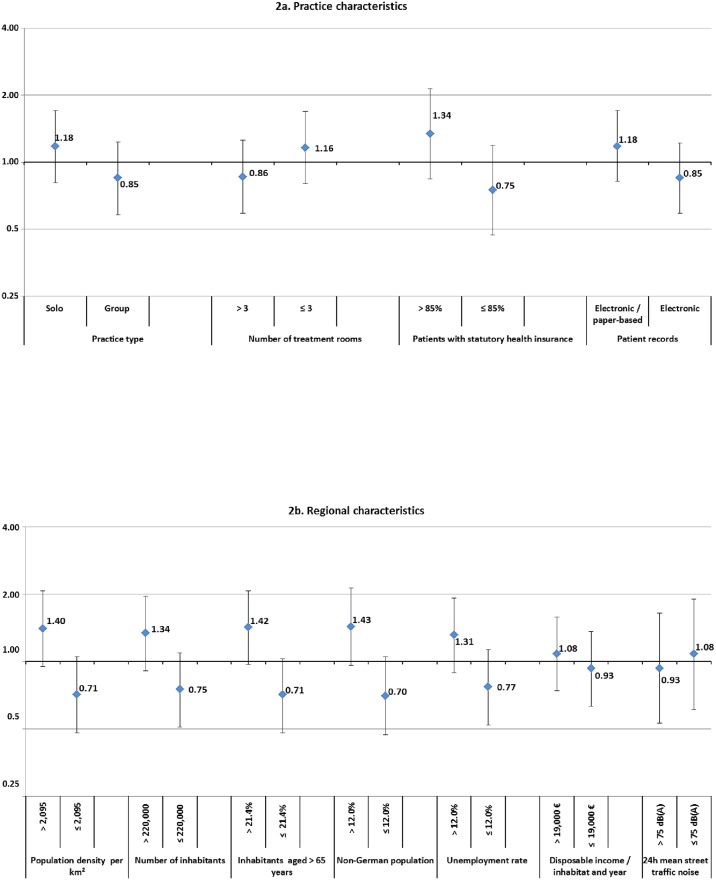
**2a. Practice characteristics**. Prevalence ratios for practices with low strain due to chronic stress (≤16.6) versus practices with high strain due to chronic stress (>16.6) by practice characteristics. **2b. Regional characteristics**. Prevalence ratios for practices with low strain due to chronic stress (≤16.6) versus practices with high strain due to chronic stress (>16.6) by regional characteristics.

There were no differences in PRs when stratifying for practice type (solo / group) (Fig 2a), while we saw a higher PR for practices serving a patient population with > 85% patients covered by statutory health insurance (PR 1.34 [95% CI 0.84–2.14]). Practices using a combination of electronic and paper-based records were 1.18 times (95% CI 0.82–1.71) more likely to be stressed than practices documenting purely electronically.

### Regional characteristics

There was no association between the seven regional characteristics analyzed (population density, number of inhabitants, proportion of inhabitants aged ≥65 years, percentage of non-German population, unemployment rate, disposable income per year and inhabitant, mean 24-hour traffic noise) and the prevalence ratios for chronic stress (Fig 2b).

## Discussion

In this sample of GPs and PAs we observed a 26.3% prevalence of high strain due to chronic stress, with the highest values seen in female GPs and PAs. Stress levels were higher in these primary care professionals than in the German general population [14]. In addition, our analysis on practice cluster level showed high intra-cluster correlations for GPs and PAs. Importantly, the ICCs calculated were much higher than those reported in a study on job satisfaction among German GPs and PAs [7] and in cluster studies from primary care addressing various patient outcomes [20].

We observed associations between personal and practice characteristics with chronic stress. However, our prevalence rates for high strain are up to two-fold higher than in the German general population according to the above-mentioned German DEGS1 which used the same study instrument (women: 26.4% in PA, 35.6% in GPs vs. 13.9% in DEGS1; men: 19.9% in GPs vs. 8.2% in DEGS1). The higher stress prevalence observed among females is in line with the general population [14]. This gender association is also reported for German hospital physicians, where females were found to have a significantly higher prevalence of stress than males (59.7% vs. 51.5%, OR 1.40 [95% CI 1.17–1.66]) [2]. In addition, female hospital physicians had a significantly lower sense of “control” [2] which may play a role in coping with stress. In the literature, several additional factors are discussed as reasons for gender differences, for example an effect related to the study instrument (TICS-SSCS) [21], biological differences [13] and gender-specific socialization. Ceccato, Kudielka and Schwieren (2016) showed higher TICS-SSCS scores in women compared to men, while the same male study participants had an opposite trend for hair cortisol measurements [21]. Across studies, challenges of work-family balance are more prevalent in females [9,22]. In contrast, the literature is inconclusive regarding the association between gender and various indicators for low physician well-being, e.g., higher rates of burnout were reported among male physicians [5], yet higher rates of depression in female physicians [9].

In addition to gender, social status was associated with the level of stress [14]. The DEGS1 reported an inverse relationship between socio-economic status and high strain among males, while this difference was not significant between females with medium and high socio-economic status [14]. Given that practice assistants are overwhelmingly females (98.5%), we were able to stratify by social status for female participants only. In agreement with the DEGS1 findings, there was no inverse relationship between socio-economic status and high strain for females, in fact we even showed higher stress among the higher socio-economic status female physicians than among practice assistants.

We observed a higher proportion of high stress in younger participants, which was reported in previous studies in primary care for burnout [5]. As female GPs in our study were younger and had less work experience than their male counterparts, this may offer another explanation for the higher stress prevalence in the female subgroup. As age and work experience are interrelated factors, associations with stress cannot be disentangled completely. In contrast to these results, a study on job satisfaction—which is considered protective against negative consequences of work-related stress [4,23]–in a German GP population observed greater satisfaction in younger physicians [24]. High satisfaction rates were seen in GPs who were satisfied with the patient contact, which is related to the time a physician has for a single consultation and the total patient caseload. Our results suggest that the caseload itself is not associated with a higher prevalence of stress, but with more working hours, which are needed to ensure an adequate time per patient. Similar relationships between having an influence on work hours and lower physician wellbeing, i.e. burnout [25], fatigue after work [25], depression [26], motor-vehicle crashes or near miss incidents when driving home [27], and failures of attention [28] are documented in various studies [9].

A previous study showed that higher job satisfaction of German practice assistants was associated with working part-time [29]. Because we also saw higher strain due to chronic stress in this very group, future studies should focus on work-time management and work-life balance.

On a practice level we did not observe differences in prevalence by practice type, which is in line with the results of Harris et al. [8]. In the study with European GPs, depersonalization (burnout) was more prevalent in GPs in group practices [5], while in a German GP population dissatisfaction was higher in physicians in single physician practices. In our analysis, the use of electronic patient records was slightly associated with lower stress, but lacked significance (small number of cases). In US primary care physicians, stress was higher in physicians from practice clusters using electronic medical records with more functions compared to those with fewer functions [30]. There was no association between regional characteristics and stress prevalence for both professional groups.

### Strength and limitations

The response rate of the practices was very high, reflecting a high interest in this topic. Other reasons might have been the data collection method (on-site visits) and the practices´ affiliation with the institute. Non-response due to reasons such as “no time” or “too much stress” may have led to an underestimation of the prevalence of stress, yet marked underestimation can be excluded given the high response rate.

Although all participating practices belong to the same practice network, a potential selection bias can be excluded as we showed in a prior study that the practice sample is representative for GP practices in Germany. The practice physician sample associated with our institute differs only with regard to the proportion of female physicians and with regard to the proportion of GPs working in group practices [10]. We addressed the first aspect by calculating gender-specific cut-offs for the stress categories. As no difference in the prevalence of chronic stress was observed by practice types, no additional adjustment was required.

## Conclusion

This study investigated chronic stress in primary care practice teams with analyses by professional group and on a practice level. The prevalence of strain due to chronic stress in these primary care workers is high, which indicates a need for stress reduction strategies/programs. High intra-cluster correlations indicate that such strategies should address practice teams by including both professional groups. Furthermore, key determinants for perceived stress should be addressed when planning stress reduction strategies. Gender aspects related to chronic stress are just as important as practice management aspects, e.g., the physicians´ caseload and working hours. Like other studies, our analysis suggests that an organization-targeted intervention can potentially improve practice management-related issues.
